# Reducing fatigue-related symptoms in Long COVID-19: finding an intervention that works

**DOI:** 10.1192/bjo.2021.681

**Published:** 2021-06-18

**Authors:** Adrian Heald, Lisa Riste, Andreas Walther, Mike Stedman, Annice Mukherjee, Ray Perrin

**Affiliations:** 1 Salford Royal Foundation Trust; 2 University of Manchester; 3 University of Zurich; 4 Res Consortium

## Abstract

**Aims:**

In the early days of the first global wave of the COVID-19 pandemic, the potential for a post-viral syndrome to manifest following COVID-19 infection was highlighted.

It was pointed out that an early intervention applying management techniques used in patients with CFS/ME appeared to help reduce the fatigue related symptoms of Long COVID.

Here we present an analysis of a consecutive case series of the first twenty patients’ data collected. Our aim was to evaluate the potential of this mode of treatment for Long COVID.

**Method:**

Face to face treatment sessions with the practitioners occurred once a week, involving effleurage and other manual articulatory techniques.

The individuals being treated also undertook a daily self-massage along with gentle mobility exercises and alternating warm and cool gel packs on the upper spine, to encourage a reduction of spinal inflammation and further aid lymph drainage of the brain and spine.

Symptom severity was recorded using the self-reported 54-item Profile of Fatigue Related States (PFRS).

**Result:**

The mean age of the men was 41.8 years with a range of 29.1-53.1 years with the corresponding mean age for women being 39.3 years with a range of 28.3-50.4 years.

The average time interval between onset of Coronavirus symptoms and start of treatment for Long COVID was just over 20 weeks. The average number of treatment sessions was similar at 9.7 in men and 9.4 in women.

The change in Profile of Fatigue Related States (PFRS) score was similar in men with a significant decrease (-45%) as in women (-52%) (F 4.8, p < 0.001).

None of the individuals had any prior diagnosis of chronic fatigue syndrome.

All were new attendees to the clinic at the time of initial assessment.

**Conclusion:**

Our findings indicate that this intervention based on massage and mobility exercises significantly reduced fatigue related to Long COVID.

It may be that early intervention and supportive treatments at the end of the acute phase of COVID-19 can help overcome acute phase symptoms and prevent them becoming chronic/enduring.

